# Supplementary material for: Routine, Cost-Effective SARS-CoV-2 Surveillance Testing Using Pooled Saliva Limits Viral Spread on a Residential College Campus
Source: Microbiol Spectr. 2021 Oct 13;9(2):e01089-21. doi: 10.1128/Spectrum.01089-21 (PMC8515933; doi:10.1128/Spectrum.01089-21)
Supplement: SUPPLEMENTAL FILE 1 — Supplemental material. Download SPECTRUM01089-21_Supp_1_seq9.pdf, PDF file, 0.5 MB [file spectrum01089-21_supp_1_seq9.pdf]

Supplementary Figure 1: Limit of SARS-CoV-2 detection and assay performance validation. (A) N1 Ct values of the positive control plasmid from each run throughout the semester. (B) Ten replicates of each of three presumed positive saliva samples were run on a single plate for evaluation of within-run consistency. (C) Two replicates of each of three presumed positive saliva samples were run on one plate each day for seven days for evaluation of between-run consistency. Means of replicate samples are indicated by black lines. (D) Plot of individual RP and N1 Ct values for flagged saliva samples (R^2=0.0036).

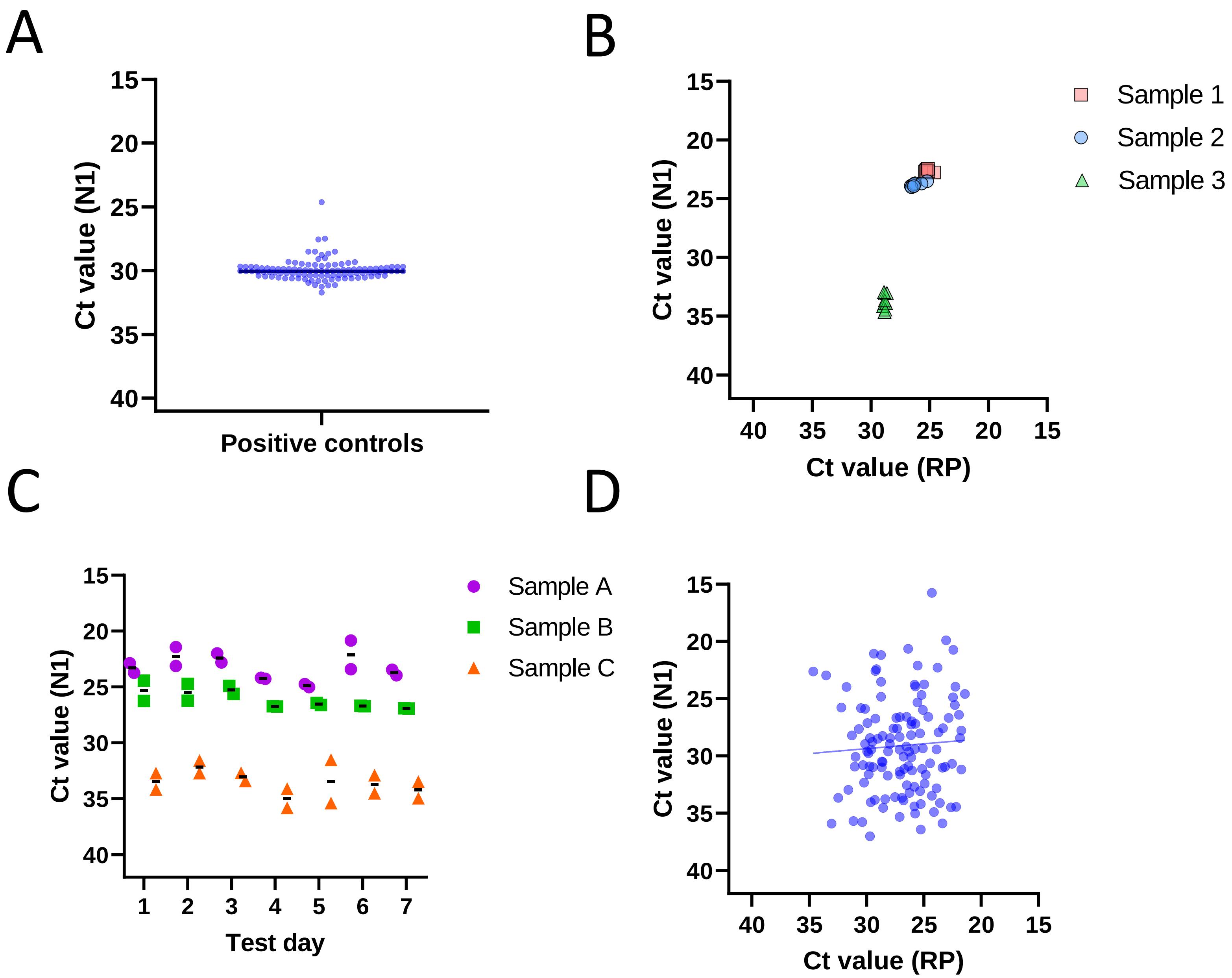

Supplementary Table 1: ONU SARS-CoV-2 RT-qPCR Cost Estimates

Company	*Item*	*Catalog #*			*Cost per Well*
Integrated DNA Technologies	nCOV_N1 Forward Primer Aliquot, 100 nmol	10006830			$0.01
	nCOV_N1 Reverse Primer Aliquot, 100 nmol	10006831			$0.01
	nCOV_N1 Probe Aliquot, 50 nmol	10006832			$0.02
	RNase P Forward Primer Aliquot, 100 nmol	10006836			<$0.01
	RNase P Reverse Primer Aliquot, 100 nmol	10006837			<$0.01
	RNase P (ATTO™ 647) Probe, 50 nmol	10007062			$0.04
ThermoFisher Scientific	TaqPath 1-Step RT-qPCR Master Mix, CG	A15300			$1.45
	EnduraPlate Optical 96-Well Clear Reaction Plates	A36924			$0.08
	Adhesive PCR Plate Seals	AB0558			$0.01
			Individual Samples	*Pools of 5*	*Pools of 10*
Midwest Scientific	PCR 8 Strip Tubes with Attached Caps, 0.2mL	PR-PCR28ACF	1 x $0.06 = $0.06	1 x $0.06 = $0.06	1 x $0.06 = $0.06
	10uL & 200uL reload tips	PR-10/200RFL	2 x $0.02 = $0.04	6 x $0.02 = $0.11	11 x $0.02 = $0.21
	Prima 1.5mL tubes	PR-MCT15	6 x $0.03 = $0.17	10 x $0.03 = $0.28	15 x $0.03 = $0.42
Labtag	Cryo Inkjet Labels	A4AJA-12	1 x $0.02 = $0.02	5 x $0.02 = $0.08	10 x $0.02 = $0.15
Reagent Total (with 10% overage)			*$1.82*	*$1.82*	*$1.82*
Materials Total			*$0.28*	*$0.52*	*$0.83*
Total Costs per Well			***$2.09***	***$2.34***	***$2.65***

Cost estimate range for pools of 5, 10, and ONU Saliva Testing†

	ONU Daily Average	*Run Individually*	*Pools of 5*	*Pools of 10*
Flagged Run	44 pools of 10 and 45 pools of 5	465-930	93	93
Deconvolution Run	31	0	0-90	0-90
Average Cost per Sample	*$0.43*	*$2.09*	*$0.47-$0.87*	*$0.27-$0.47*
Total Daily Screening Cost	***$286***	***$972-$1,944***	***$218-$406***	***$247-$435***

†Assumes one flagged run and one deconvolution run where pools of 10 reach maximum capacity with 9 positives among 93 pools (930 samples), while pools of 5 reach maximum capacity with 18 positives among 93 pools (465 samples)

Supplementary Table 2: Saliva testing participants

	Individuals	Submitted Tests
Required Students	2003	41463
2x week	1210	
1x week	793	
Voluntary	214	2421
Employees	182	
Commuters	32	
Totals	*2217*	*43884*

Supplementary Table 3: Pools of five and ten flag positives at expected intervals†

	Total Pools (%)	Total Tests (%)	Positive Pools (%)
Pools of 10	2787 (49.8%)	27870 (66.8%)	62 (62.6%)
Pools of 5	2466 (44.1%)	12330 (29.6%)	33 (33.3%)
Pools of other	344 (6.1%)	1515 (3.6%)	4 (4%)

†Semester testing here excludes intake testing

Supplementary Table 4: Characteristics of COVID-19 survey respondents & ONU's student body

	Characteristics of Survey Respondents			Characteristics of Student Body	
	Number	Percentage of Survey Respondents		Number	Percentage of Student Body
Survey Respondents	66		Total Student Body	2343	
Age					
Ages 18-25	66	100.0%		2265	96.7%
Sex/Gender					
Female	41	62.1%		1327	56.6%
Male	25	37.9%		1016	43.4%
Types of Students					
Residential	64	97.0%		1893	80.8%
Commuter	2	3.0%		450	19.2%
Ethnicity					
White	59	89.4%		1728	73.8%
Asian	1	1.5%		57	2.4%
Black or African American	1	1.5%		161	6.9%
Hispanic or Latino	3	4.6%		265	11.3%
Other	1	1.5%		124	5.3%
I prefer not to answer (or none provided)	1	1.5%		8	0.3%
Pre-existing health status					
No known pre-existing health conditions	54	81.8%			
Asthma	7	10.6%			
Obesity	2	3.0%			
Other health conditions	3	4.5%			
Preferred not to answer	1	1.5%			

Supplementary Table 5: Accuracy of first diagnostic test results for COVID-19-positive individuals

Ct value (N1) of saliva	Number of first diagnostic tests* that were positive	Number of students	Percent accuracy of first diagnostic test
**20-29.99**	29	32	90.63%
20-21.99	2	2	100.00%
22-23.99	5	5	100.00%
24-25.99	6	7	85.71%
26-27.99	9	9	100.00%
28-29.99	7	9	77.78%
**30-35.99**	20	29	68.97%
30-31.99	12	15	80.00%
32-33.99	6	10	60.00%
34-35.99	2	4	50.00%
**All Ct values**	49	61	80.33%

*Note that the first diagnostic test was typically administered 24-48 hrs after the flagged saliva test.

COVID-19 Research Survey

Prelude to survey:

We hope that our campus's experience with COVID-19 will help nursing homes and other organizations better manage viral spread and ultimately save lives. Your accurate and truthful answers are critical to accomplishing this mission; please use integrity when completing this survey. The outcomes of this research will be reported anonymously and in aggregate so that no individuals could be identified based on the reported data.

Survey questions:

1. ONU recently detected evidence of SARS-CoV-2 (the COVID-19 virus) in your barcoded saliva sample. After you were notified of this, did you subsequently test positive for COVID-19 via any additional diagnostic tests (such as from Health Services or a hospital)?
   - ☐ Yes
   - ☐ No

2. Please describe the additional COVID-19 testing that you received AFTER your saliva tested positive. For EACH test, please answer as many of the following as you can:

   WHAT type of test was performed? (This should be indicated on your testing results paperwork. Examples include "Sofia 2 SARS Antigen FIA", “Abbott BinaxNOW”, "PCR (or RNA) test", or you may state “unsure”.)
   WHEN was the test performed?
   WHERE was the test performed?
   WHAT was the result?

   Example: After my saliva tested positive, I got a rapid antigen test from Health Services on 1/20/21. That came back negative, so they told me to wait 48 hours and get tested again. On the second rapid antigen test from them on 1/22/21, the test came back positive.

   a. Open textbox for responses

3. During the past approximately 10-15 days, which of the following symptoms did you experience? Select all that apply, excluding symptoms that you regularly live with (such as allergy symptoms).
   - ☐ Fever or chills
   - ☐ Cough
   - ☐ Sore throat
   - ☐ Congestion or runny nose
   - ☐ Muscle or body aches
   - ☐ Headache
   - ☐ Loss of taste or smell

- ☐ Difficulty breathing or shortness of breath
- ☐ Fatigue
- ☐ Nausea or vomiting
- ☐ Diarrhea
- ☐ I did not experience any new symptoms
- ☐ Other: _________________ (text box)

4. Were you experiencing any of the above symptoms at the time of your positive saliva sample submission?
   - ☐ Yes, very noticeable symptoms.
   - ☐ Yes, but very mild symptoms.
   - ☐ No, I was not experiencing symptoms at that time.
   - ☐ I don't remember.

5. As best as you can recall, when did your symptoms (if any) begin? If you cannot remember or did not experience symptoms, you may skip this question.
   a. Dropdown calendar to select date (M/d/yyyy)

6. How would you describe the severity of your symptoms (if any)?
   - ☐ Very severe (required hospitalization)
   - ☐ Severe (required treatment from a doctor)
   - ☐ Moderate (flu-like symptoms that were managed at home)
   - ☐ Mild (minor discomfort such as loss of taste/smell or like a common cold)
   - ☐ Asymptomatic (did not experience symptoms)

7. Have you previously tested positive for COVID-19 via a "nasal" test? (Only include official diagnoses.)
   - ☐ Yes
   - ☐ No

8. (If yes to 7) When did you previously test positive for COVID-19 via a "nasal" test?
   a. Dropdown calendar (M/d/yyyy)

9. Have COVID-19 antibodies previously been detected in your blood?
   - ☐ Yes.
   - ☐ No, I was tested but no antibodies were detected.
   - ☐ I have not been tested for antibodies in my blood.

10. (If yes to 9) What is the most recent date that antibodies were detected in your blood?
    a. Dropdown calendar (M/d/yyyy)

11. Have you been vaccinated against COVID-19?
    - ☐ Yes
    - ☐ No

12. (If yes to 11) Please describe WHEN you received your vaccine dose(s) and WHICH vaccine you received (if you know).

    Example: My first dose was on 12/20/21. Second dose was 1/15/21. I received the Pfizer vaccine.

    a. Open textbox for responses

13. As best as you can recall, how many "close contacts" did you have within the 48 hours prior to submitting your positive saliva sample? A close contact is defined as someone who was within 6 feet of you for a total of 15 minutes. (These are the individuals whom you should have reported to the Health Services contact tracing team.)
    a. Dropdown menu of number options

14. As best as you can recall, of your close contacts (as identified in the previous question), how many have since tested positive for COVID-19, either by the university's saliva-based test and/or a diagnostic COVID-19 test? Do not include any individuals of whose results you are uncertain.
    a. Dropdown menu of number options

15. Of those that tested positive, describe your interaction with each close contact during the 48 hours prior to submitting your positive saliva sample. Separate each individual by a number. Please include answers to each of these questions in your description:

    How LONG were you in contact?
    How CLOSE were you to the person?
    Were you wearing MASKS?

    For example…

    Positive contact 1: 30-minute unmasked car ride

    Positive contact 2: I had a 1-hr basketball practice with this contact where we wore masks. Then we ate dinner together in Ludwig (no masks). Then we watched a 2-hr movie together while wearing masks and sat 3 feet apart.

    a. Open textbox for responses

16. As best as you can recall, how many of your close contacts have since tested negative for COVID-19, either by the university's saliva-based test and/or a diagnostic COVID-19 test? Do not include any individuals of whose results you are uncertain.
    a. Dropdown menu of number options

17. If one of your close contacts was a roommate, has he/she continued to test negative for COVID-19, either by the university's saliva-based test and/or a diagnostic COVID-19 test, after being exposed to you?
    - ☐ Yes, he/she has continued to test negative.
    - ☐ No, he/she has tested positive.
    - ☐ I don’t have a roommate (or they weren’t considered a close contact).
    - ☐ I don’t know.
    - ☐ Other: _________________ (text box)

18. Which of the following best describes your association with the university?
    - ☐ Residential student (live in university dorms or apartments)
    - ☐ Commuter student
    - ☐ Staff
    - ☐ Full-time faculty
    - ☐ Adjunct faculty

19. What is your approximate age?
    - ☐ 18-25
    - ☐ 26-30
    - ☐ 31-39
    - ☐ 40-49
    - ☐ 50-59
    - ☐ 60-69
    - ☐ >70
    - ☐ I prefer not to answer

20. What is your gender?
    - ☐ Male
    - ☐ Female
    - ☐ I prefer not to answer

21. What is your race?
    - ☐ White
    - ☐ Black or African American
    - ☐ Hispanic or Latino
    - ☐ Asian
    - ☐ I prefer not to answer
    - ☐ Other: _________________ (text box)

22. Do you have pre-existing health conditions? (Select all that apply.)
    - ☐ Diabetes
    - ☐ Asthma
    - ☐ Immunocompromised
    - ☐ Obesity
    - ☐ No known pre-existing health conditions
    - ☐ I prefer not to answer
    - ☐ Other: _________________ (text box)

23. Thank you for taking the time to complete this survey! We hope that our campus's experience with COVID-19 will help nursing homes and other organizations better manage viral spread and ultimately save lives. Your accurate and truthful answers in this survey are critical to accomplishing this mission. Please verify below that your answers are truthful and accurate to the best of your knowledge.
    - ☐ Yes, I completed this survey with integrity.
    - ☐ No, my data may not be truthful or accurate.
